# Thiazolidin-2-cyanamides derivatives as novel potent *Escherichia coli* β-glucuronidase inhibitors and their structure–inhibitory activity relationships

**DOI:** 10.1080/14756366.2020.1816998

**Published:** 2020-09-14

**Authors:** Tao-Shun Zhou, Bin Wei, Min He, Ya-Sheng Li, Ya-Kun Wang, Si-Jia Wang, Jian-Wei Chen, Hua-Wei Zhang, Zi-Ning Cui, Hong Wang

**Affiliations:** a College of Pharmaceutical Science and Collaborative Innovation Center of Yangtze River Delta Region Green Pharmaceuticals, Zhejiang University of Technology, Hangzhou, China; b Center for Human Nutrition, David Geffen School of Medicine, University of California, Los Angeles, CA, USA; c State Key Laboratory for Conservation and Utilization of Subtropical Agro-Bioresources, Lingnan Guangdong Laboratory of Modern Agriculture, Integrative Microbiology Research Centre, Guangdong Province Key Laboratory of Microbial Signals and Disease Control, South China Agricultural University, Guangzhou, China

**Keywords:** Structure–inhibitory activity relationships, thiazolidin-2-cyanamide derivatives, *Escherichia coliβ*-glucuronidase, *β*-glucuronidase inhibitors, CPT-11-induced toxicity

## Abstract

Gut microbial β-glucuronidases have the ability to deconjugate glucuronides of some drugs, thus have been considered as an important drug target to alleviate the drug metabolites-induced gastrointestinal toxicity. In this study, thiazolidin-2-cyanamide derivatives containing 5-phenyl-2-furan moiety (**1–13**) were evaluated for inhibitory activity against *Escherichia coli* β-glucuronidase (EcGUS). All of them showed more potent inhibition than a commonly used positive control, d-saccharic acid 1,4-lactone, with the IC_50_ values ranging from 1.2 µM to 23.1 µM. Inhibition kinetics studies indicated that compound **1**–**3** were competitive type inhibitors for EcGUS. Molecular docking studies were performed and predicted the potential molecular determinants for their potent inhibitory effects towards EcGUS. Structure–inhibitory activity relationship study revealed that chloro substitution on the phenyl moiety was essential for EcGUS inhibition, which would help researchers to design and develop more effective thiazolidin-2-cyanamide type inhibitors against EcGUS.

## 1. Introduction

Hepatic glucuronidation is an important process of drug detoxication and a major pathway of phase II xenobiotic biotransformation in the human body^1–3^. However, many drugs can be excreted into the intestinal lumen after they are glucuronidated and usually can be reactivated by gut microbial β-glucuronidases. Unfortunately, sometimes the reactivation can cause severe gastrointestinal damage, for example, hydrolysis of metabolites from irinotecan (CPT-11) or carboxylic acid-containing non-steroidal anti-inflammatory drugs (NSAIDs) lead to severe late diarrhoea in patients receiving therapy^4–7^.

Interestingly, recent studies demonstrated that gut microbial β-glucuronidase inhibitors, such as *Escherichia coli* β-glucuronidase (EcGUS) inhibitors, could significantly attenuate gastrointestinal toxicity caused by CPT-11 and NSAIDs^8^^,^^9^. With the deepening of research on gut microbial β-glucuronidase inhibitors, more and more natural and synthetic EcGUS inhibitors have been reported^10^. Previous studies reported many synthetic EcGUS inhibitors, for example, an antidepressant, amoxapine, was identified as a potent EcGUS inhibitor^11^, Salar et al.^12^ evaluated the inhibitory effects of twelve thiadiazole derivatives towards EcGUS with IC_50_ values ranging from 3.10 µM to 35.40 µM, Taha et al.^13^ reported that oxadiazole coupled-thiadiazole derivatives as potent EcGUS inhibitors and the most active inhibitor with an IC_50_ value of 0.96 µM. Interestingly, all these types of compounds contain an extremely similar moiety, a phenyl group substituted with a heterocycle. Molecular docking studies further demonstrated that both the phenyl and heterocyclic groups interacted with the corresponding pocket residues via π–π stacking, and the heterocyclic nitrogen, sulphur and/or oxygen increased hydrogen bonding capability of these compounds for pocket binding^14–16^. The structure of 5-phenyl-2-furan is very similar to the above mentioned structural units. Additionally, our previous studies have reported that the derivatives of 5-phenyl-2-furan showed broad-spectrum bioactivities, such as antibacterial, antitumor, and anti-inflammatory activities^17–20^. In 2018, we synthesised a series of thiazolidin-2-cyanamide derivatives, which also contained 5-phenyl-2-furan moiety and could reduce the disease symptoms of *Xanthomonas oryzae* pv. *oryzae* on the rice cultivar IR24^21^.

Therefore, in this study, 13 thiazolidin-2-cyanamide derivatives containing 5-phenyl-2-furan moiety were selected and subjected to evaluate their inhibitory effects on EcGUS. *p*-Nitrophenyl-β-d-glucuronide acid (PNPG) was used as the probe substrate for EcGUS. Then the inhibitory behaviours of these compounds against EcGUS were characterised and their structure–inhibitory activity relationships were explored. Finally, molecular docking studies were performed to predict the molecular determinants of thiazolidin-2-cyanamide derivatives against EcGUS.

## 2. Materials and methods

### 2.1. Materials

Thirteen thiazolidin-2-cyanamide derivatives containing 5-phenyl-2-furan moiety were provided by Prof. Zi-Ning Cui from South China Agricultural University (Guangzhou, China). *p*-Nitrophenyl-β-d-glucuronide acid (PNPG), d-saccharic acid-1,4-lactone (DSL), and dimethyl sulfoxide (DMSO) were supplied by Sigma-Aldrich (St. Louis, MO, USA). Dulbecco’s phosphate-buffered saline (PBS) was provided by Life Technologies (Carlsbad, CA, USA). Imidazole, kanamycin, and isopropyl β-d-1-thiogalactopyranoside (IPTG) were supplied by Biosharp (Hefei, China). Recombinant *E. coli* BL21 (DE3) harbouring pET28a-EcGUS was generously provided by Prof. Ru Yan from the University of Macau (Macau, China). Deionised water was purified by a Milli-Q purification system (Millipore, Bedford, MA, USA). Purities were all >98%.

### 2.2. General synthetic procedure

**Figure 1.**
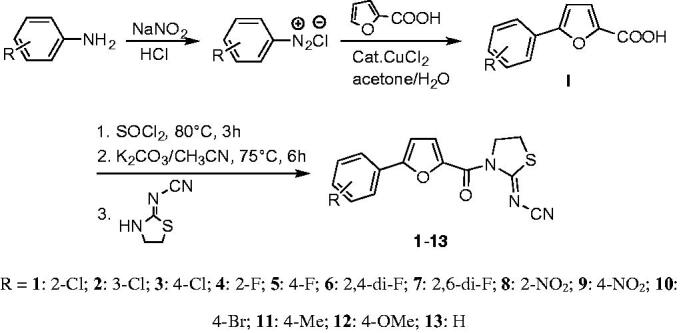
General synthetic procedure for title compounds **1**–**13**. R = 1: 2-Cl; 2: 3-Cl; 3: 4-Cl; 4: 2-F; 5: 4-F; 6: 2,4-di-F; 7: 2,6-di-F; 8: 2-NO_2_; 9: 4-NO_2_; 10: 4-Br; 11: 4-Me; 12: 4-OMe; 13: H.

**Figure 2.**
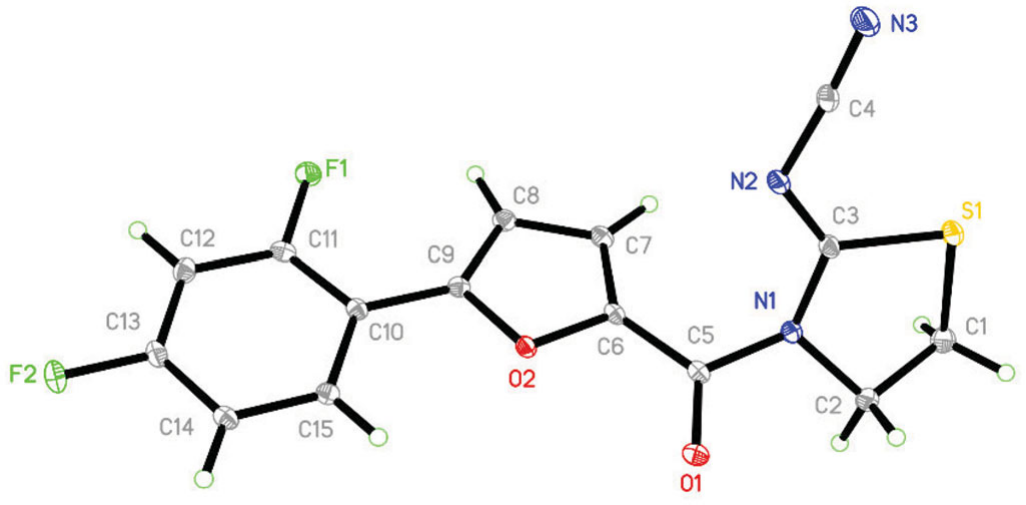
Single crystal structure of compound **6**.

The synthetic route of title compounds was shown in Figure 1. The key intermediate I was synthesised from substituted aniline by Meerwein arylation reaction according to the reported procedure^22^^,^^23^. A mixture of 5-substituted phenyl-2-furancarboxylic acid I and thionyl chloride was refluxed in anhydrous toluene at 80 °C for 3 h to afford the 5-phenyl-2-furancarbonyl chloride, which was added into 2-cyanoiminoradical-1, 3-thiazolidine in refluxing anhydrous acetonitrile in presence of an equivalent amount of potassium carbonate at 75 °C for 3–6 h to afford the title compounds in moderate or good yields (for the details, see Supplementary Materials). The structures were also further confirmed by X-ray single-crystal analysis and a perspective view of the compound **6** (CCDC No.: 1565820) was shown in Figure 2.

### 2.3. Enzyme preparation

EcGUS was prepared according to our previous report^24^. The recombinant strain were incubated in 200 mL of LB broth (Tryptone, 10 g/L; yeast extract, 5 g/L; NaCl, 10 g/L; pH 7.0) containing 1% kanamycin at 220 rpm and 37 °C until OD600 reached 0.5–0.6. Afterward, IPTG (final concentration, 0.5 mM) was added and the culture was incubated at 220 rpm and 16 °C for 16 h to induce the expression of β-glucuronidase. The cells were collected by centrifugation and suspended in PBS buffer (pH 7.4), and then applied to extract the enzyme by sonication. Finally, pure EcGUS was obtained from the cell-free extracts through a Ni-NTA column (EMD Millipore Corp., MA, USA). Protein concentration was determined using the BCA Protein Assay Kit (Beyotime, Shanghai, China) according to the manufacturer’s instruction, and its purity was determined by SDS-PAGE.

### 2.4. Enzyme inhibition assays

Thirteen thiazolidin-2-cyanamide derivatives were subjected to screening for potent EcGUS inhibitor. The inhibitory effects of these compounds were determined by measuring the PNP formation generated from PNPG by EcGUS. Briefly, the assays were conducted in 96-well flat-bottomed tissue culture plates (Nunc, Denmark) with a total volume of 100 µL which consisted of 10 µL EcGUS (final concentration, 2 µg/mL), 70 µL PBS buffer (pH 7.4), 10 µL test compound or DSL (final concentration, 10 µM) and 10 µL PNPG (final concentration, 250 µM). DSL was used as a positive control, and 1%DMSO was used as blank control. All reactions were performed in triplicate and the produced PNP was monitored by measuring absorbance at 405 nm in a plate reader (SpectraMax Plus384, Molecular Devices, CA, USA) after incubating at 37 °C for 30 min. The PNP concentration was determined from a standard curve (0, 10, 20, 40, 60, 80, 100, 120 µM PNP) in PBS buffer (pH 7.4). Relative activity was defined as a percent of PNP formation in the presence of test compound within 30 min compared with the blank control group^25^.

### 2.5. Inhibition kinetic analysis

IC_50_ values of the 13 thiazolidin-2-cyanamide derivatives against EcGUS were investigated using similar assays as described in Section 2.4. The reaction conditions are as follows: 10 µL pure enzyme (final concentration, 2 µg/mL), 70 µL PBS buffer (pH 7.4), 10 µL test compound or DSL (final concentration, 0.001–5000 µM) and 10 µL PNPG (final concentration, 250 µM) incubating at 37 °C for 30 min. The IC_50_ values were calculated by plotting the concentration–response curve using GraphPad Prism 6.0 (GraphPad Software, La Jolla, CA).

To further characterise the inhibition behaviour of discovered inhibitors, multiple concentrations of compounds (0.5–12 µM) or DSL (50–100 µM) and PNPG (0.2, 0.3, 0.5, and 1.0 mM) were applied to determine the corresponding reaction rates. The inhibition type was evaluated through determining the intersection point in Lineweaver–Burk plots, and the K_i_ values were calculated by nonlinear regression of different inhibition types using the GraphPad Prism 6.0 (GraphPad Software, La Jolla, CA) ^25–27^.

### 2.6. Molecular docking studies

Molecular docking studies were conducted to explore the molecular determinants of discovered inhibitors against EcGUS using MOE (Version 2014. 09, Chemical Computing Group Inc., Montreal, Canada). Docking studies were performed according to our previous report^25^. Briefly, the X-ray crystal structure of EcGUS (PDB ID: 3K4D) was retrieved from the Protein Data Bank and prepared using MOE. Each inhibitor was docked into the active site of EcGUS using the Triangular Matching docking method and 30 conformations of each ligand–protein complex were generated according to the docking score. Docking poses were visually inspected to analyse the interactions of inhibitors with binding pocket residues.

### 2.7. Statistical analysis

All experiments were carried out in triplicate and repeated at least two times. All data were expressed as mean ± standard deviation (SD). The IC_50_ values were defined as the concentration of inhibitor that is required for 50% inhibition and evaluated by nonlinear regression using GraphPad Prism 6.0 software (GraphPad Software, La Jolla, CA). The inhibition type was determined according to the intersection location of Lineweaver–Burk plot: competitive inhibition, the intersection at the y-axis; uncompetitive inhibition, parallel lines; non-competitive inhibition, the intersection at the x-axis; mixed inhibition, the intersection in first or second quadrant^28^.

## 3. Results

### 3.1. Screening of potent EcGUS inhibitor

The inhibitory effects of 13 thiazolidin-2-cyanamide derivatives were determined and presented in Table 1. Among 13 compounds (compound **1**–**13**) tested, all of them showed more potent inhibitory effects against EcGUS than DSL at 10 µM, a known positive control. Compound **2** showed the highest inhibition towards EcGUS with the inhibition rate of 95.2 ± 0.3%, followed by compounds **3** and **1**, whose inhibition rates were 80.2 ± 1.8% and 77.2 ± 0.6%, respectively (Table 1). The inhibition rates of compounds **4**–**13** ranged from 31.4% to 68.7%, all larger than that of DSL (11.6 ± 0.6%; Table 1).

**Table 1.** Chemical structures of 13 thiazolidin-2-cyanamide derivatives and the inhibitory activity against EcGUS-mediated PNPG hydrolysis.

No	R	Molecular weight (Da)	Inhibition rate at 10 μM	IC_50_ (μM)	K_i_ (μM)	Inbibition type

1	2-Cl	331.77	77.2 ± 0.6%	5.7 ± 0.6	3.5 ± 0.3	Competitive
2	3-Cl	331.77	95.2 ± 0.3%	1.2 ± 0.2	0.7 ± 0.1	Competitive
3	4-Cl	331.77	80.2 ± 1.8%	4.7 ± 0.6	2.0 ± 0.4	Competitive
4	2-F	315.32	61.6 ± 0.6%	11.9 ± 1.5	ND	ND
5	4-F	315.32	66.5 ± 1.2%	7.3 ± 0.7	ND	ND
6	2,4-di-F	333.31	43.9 ± 0.7%	23.1 ± 1.5	ND	ND
7	2,6-di-F	333.31	64.1 ± 1.2%	9.3 ± 1.1	ND	ND
8	2-NO_2_	342.33	31.4 ± 1.8%	6.1 ± 0.2	ND	ND
9	4-NO_2_	342.33	40.3 ± 3.2%	22.2 ± 2.5	ND	ND
10	4-Br	376.23	68.7 ± 1.8%	10.8 ± 0.2	ND	ND
11	4-Me	311.36	33.5 ± 2.5%	22.3 ± 2.6	ND	ND
12	4-OMe	327.36	49.6 ± 0.6%	18.5 ± 2.1	ND	ND
13	H	297.33	61.8 ± 1.3%	10.7 ± 0.5	ND	ND
d-Saccharic acid 1,4-lactone		210.14	11.6 ± 0.6%	67.3 ± 0.6	30.9 ± 2.2	ND

ND: not detect.

### 3.2. IC_50_ values of thiazolidin-2-cyanamide derivatives against EcGUS

**Figure 3.**
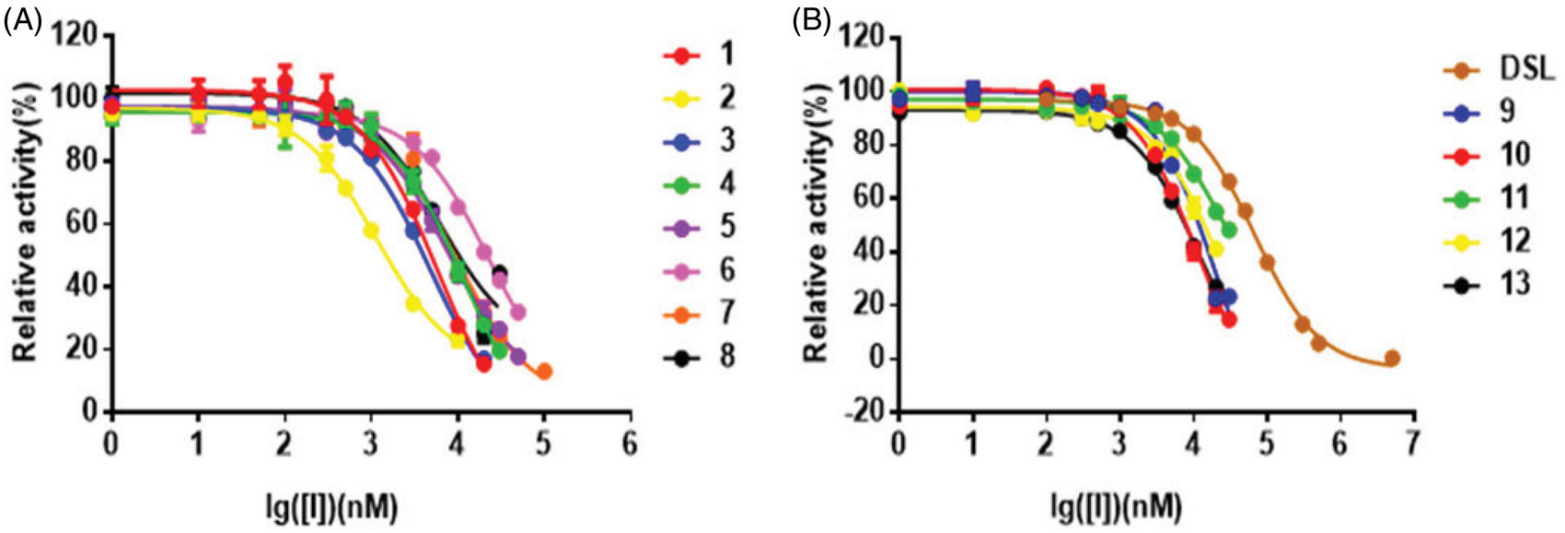
The dose-dependent inhibition curves of inhibitors on PNPG-hydrolyzing activity of EcGUS. (A) Compounds **1**–**8**; (B) compounds **9**–**13**, and DSL. All data were expressed as mean ± standard deviation of triplicate reactions.

The effects of the concentrations of the inhibitors on PNPG-hydrolyzing activity of EcGUS were presented in the dose-dependent inhibition curves. The curves confirmed all 13 thiazolidin-2-cyanamide derivatives displayed the more potent inhibitory effects on EcGUS with IC_50_ values ranging from 1.2 µM to 23.1 µM than DSL (IC_50_ = 67.3 ± 0.6 µM; Figure 3). Among them, compounds **2** and **6** displayed the most and worst potent inhibition against EcGUS with IC_50_ values evaluated as 1.2 ± 0.2 µM and 23.1 ± 1.5 µM, respectively. The IC_50_ values of compounds **1**, **3**, **5**, **7**, and **8** were less than 10 µM, suggesting that they are potent EcGUS inhibitors.

### 3.3. Structure–inhibitory activity relationships of thiazolidin-2-cyanamide derivatives against EcGUS

As shown in Table 1, all thiazolidin-2-cyanamide derivatives, compounds **1**–**13**, have different substituted phenyl moiety and demonstrated varied inhibitory potency against EcGUS. For example, compounds **2** with a meta-chloro-substituted phenyl moiety (IC_50_ = 1.2 ± 0.2 µM) showed more potent inhibition against EcGUS than compound **1** and **3** (ortho-chloro-substitution or para-chloro-substitution on the phenyl moiety, IC_50_ = 5.7 ± 0.6 µM and 4.7 ± 0.6 µM), and compound **13** (no substitution on the phenyl moiety, IC_50_ = 10.7 ± 0.5 µM), suggesting that the chloro substitution on the phenyl moiety was essential for the potent inhibition of EcGUS and meta-chloro-substitution was more beneficial than ortho-chloro-substitution or para-chloro-substitution for EcGUS inhibition.

It also can be seen from Table 1 that compound **3** with a para-chloro-substituted phenyl moiety (IC_50_ = 4.7 ± 0.6 µM) ranked the most potent inhibitor among compounds with a para-substituted phenyl moiety, including compound **5** (IC_50_ = 7.3 ± 0.7 µM), **9** (IC_50_ = 22.2 ± 2.5 µM), **10** (IC_50_ = 10.8 ± 0.2 µM), **11** (IC_50_ = 22.3 ± 2.6 µM), **12** (IC_50_ = 18.5 ± 2.1 µM), and **13** (IC_50_ = 10.7 ± 0.5 µM), indicating that effect of substitution group on EcGUS inhibition are as follows: Cl > F > Br ≈ H > OMe > NO_2_ ≈ Me. In other words, chloro and fluorine substitutions at the para position of the phenyl moiety are beneficial for EcGUS inhibition, while methoxy, methyl and nitro group substitutions are unbeneficial.

Similarly, after comparing the difference in structure and inhibitory activity of compounds **1**, **4**, **8**, and **13**, it could be easily found that chloro substitution at the ortho position of the phenyl moiety is beneficial for EcGUS inhibition, while fluorine substitution is unfavourable. Interestingly, the IC_50_ values of compound **5** (para-fluorine substitution on the phenyl moiety), **7** (2,6-difluorine substitution on the phenyl moiety), **4** (ortho-fluorine substitution on the phenyl moiety), and **6** (2,4-difluorine substitution on the phenyl moiety) were determined as 7.3 ± 0.7 µM, 9.3 ± 1.1 µM, 11.9 ± 1.5 µM and 23.1 ± 1.1 µM, respectively, demonstrating that para-fluorine substitution is more beneficial than ortho-fluorine substitution on the phenyl moiety for EcGUS inhibition, and fluorine substitution at the 2-position of the phenyl moiety of compound **5** is unfavourable for EcGUS inhibition, while fluorine substitution at the 6-position of the phenyl moiety of compound **4** is beneficial for EcGUS inhibition.

### 3.4. *Inhibition* behaviours of thiazolidin-2-cyanamide derivatives against EcGUS

**Figure 4.**
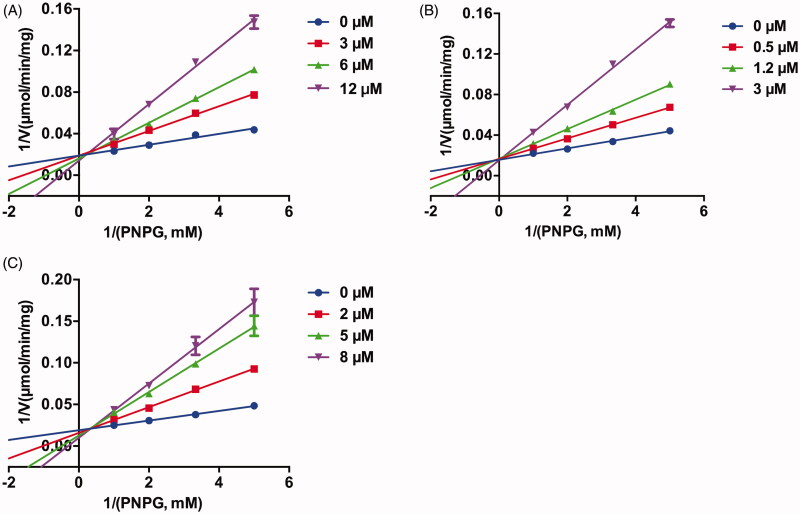
The Lineweaver–Burk plots of (A) compound **1**, (B) compound **2,** and (C) compound **3** against EcGUS. All data were expressed as mean ± standard deviation of triplicate reactions.

The inhibition behaviours of three potent EcGUS inhibitors, compounds **1**, **2**, **3**, and DSL were further investigated. As shown in Figure 4, Lineweaver–Burk plots indicated that compounds **1**, **2**, **3** were competitive inhibitors for EcGUS as judged from the intersection point at the y positive axis, implying that the binding areas of these compounds on EcGUS may be overlapped with the binding area of the substrate, PNPG, while DSL was mixed inhibitors for EcGUS as judged from the intersection point at the second quadrant. Besides, the K_i_ values of compounds **1**, **2**, **3**, and DSL also were determined as 3.5 µM, 0.7 µM, 2.0 µM, and 30.9 µM, respectively (Table 1). In summary, the results demonstrated that compounds **1**, **2** and **3** can be as potent competitive inhibitors against EcGUS with K_i_ values less than 3.5 µM.

### 3.5. Molecular docking simulations

**Figure 5.**
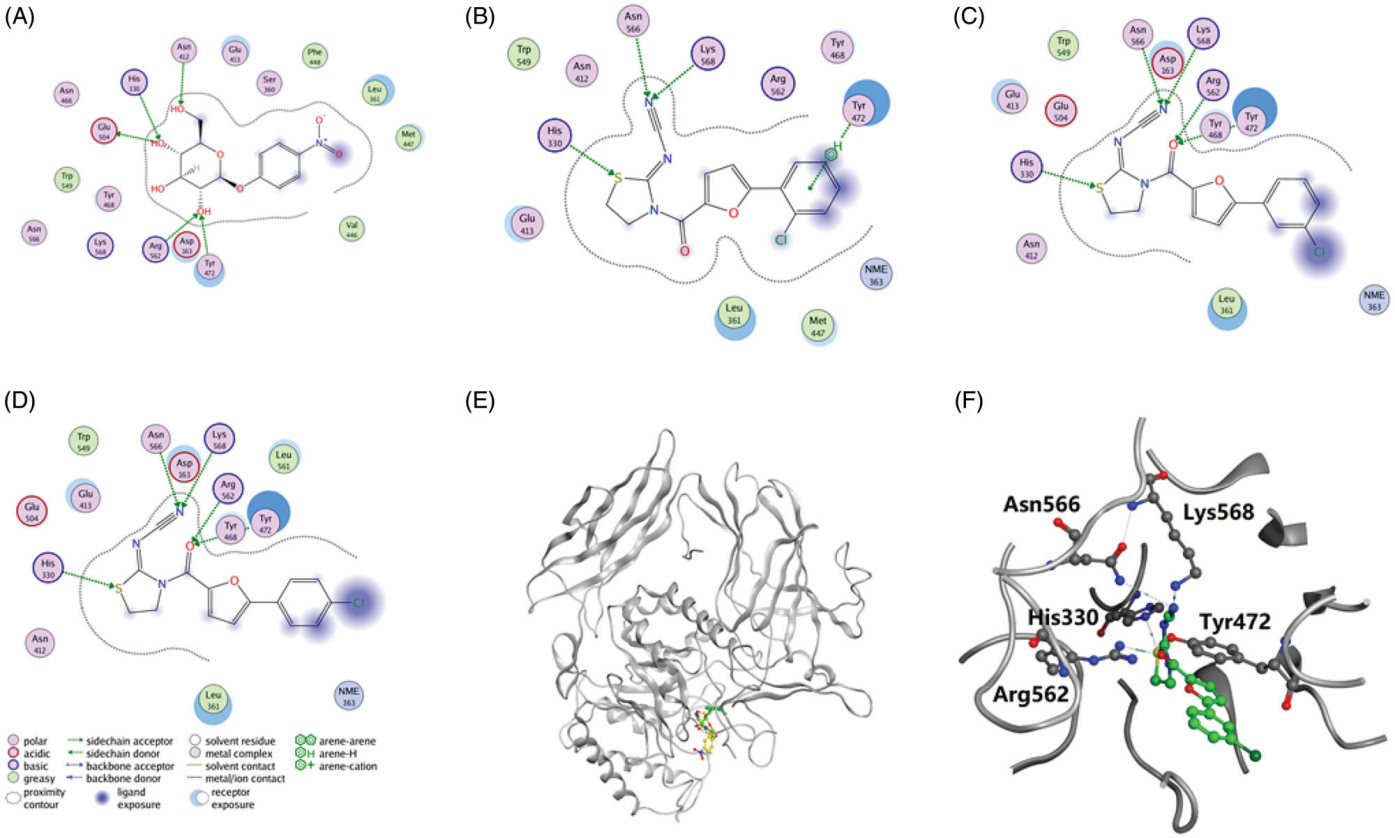
Ligand interactions of (A) PNPG, (B) compound **1**, (C) compound **2** and (D) compound **3** with EcGUS. (E) Stereo diagram of PNPG combined with compound **2** in the active site of EcGUS. (F) Binding mode of compound **2** in the active site of EcGUS.

To predict the molecular determinants of thiazolidin-2-cyanamide derivatives **1**–**3** in inhibiting EcGUS, molecular docking studies were performed using MOE. As shown in Figure 5, PNPG, compound **1**, **2**, and **3** could be well docked into the active site of EcGUS. PNPG formed hydrogen bond interactions with His330, Asn412, Tyr472, Glu505 and Arg562 (Figure 5(A)), while hydrogen bonds for compound **1** were found with residues His330, Tyr472, Asn566 and Lys568, and the binding interactions were formed with the phenolic or thiazolidine groups (Figure 5(B)). Ligand interactions between compounds **2** and **3** with EcGUS were nearly the same to each other, and also very similar to that of compound **1**, except that one more hydrogen bond interaction was observed in compounds **2** and **3**. In detail, the binding area of compound **2** was highly overlapped with that of PNPG (Figure 5(D)), and it developed five hydrogen bonds with catalytic residues His330, Tyr472, Arg562, Asn566 and Lys568 (Figure 5(C,F)), which may be associated with the more potent inhibitory potential than compund **1**.

## 4. Discussion

Gut bacterial β-glucuronidases have been proved to play important roles in CPT-11-induced intestinal toxicity, and EcGUS inhibitors have shown great potential in alleviating the toxicity. Previously identified EcGUS inhibitors usually contain heterocyclic moiety and compounds with a furan ring frequently show various bioactivities. Therefore, we evaluated the inhibitory activities of our previous synthesised thiazolidin-2-cyanamide derivatives containing 5-phenyl-2-furan moiety towards EcGUS and revealed that 13 synthetic thiazolidin-2-cyanamide derivatives were potent EcGUS inhibitors with IC_50_ values ranging from 1.2 µM to 23.1 µM, which were much more potent than a positive control, d-saccharic acid 1,4-lactone. Structure–activity relationship analysis suggested that chloro and fluorine substitutions at the para position of the phenyl moiety in thiazolidin-2-cyanamide derivatives are beneficial for EcGUS inhibition, while methoxy, methyl and nitro group substitutions are unbeneficial. Inhibition behaviour analysis indicated that compounds **1**, **2**, and **3** were identified as competitive EcGUS inhibitors with K_i_ values less than 3.5 µM and the potential molecular determinants were predicted by molecule docking studies.

Recently, 49 thiadiazole derivatives were synthesised and 38 of them were evaluated as potent bovine liver β-glucuronidase inhibitors with IC_50_ values ranging from 2.16 ± 0.01 µM to 58.06 ± 1.60 µM by Khan’s research group. Structure–activity relationship analysis also indicated that chloro substitution at the phenyl moiety in thiadiazole derivatives is beneficial for β-glucuronidase inhibition^29–31^. Since EcGUS shared high sequence similarity with bovine liver β-glucuronidase^32^ and our synthetic thiazolidin-2-cyanamides derivatives also exhibited high similarity in structure with thiadiazole derivatives, we predicted that thiazolidin-2-cyanamides derivatives may also show potent inhibition towards bovine liver β-glucuronidase and thiadiazole derivatives may be potential EcGUS inhibitors, which warrant further studies.

In the present study, we focus on investigating the effects of different substitution groups on the phenyl moiety of the thiadiazole-2-cyanamide derivatives on EcGUS inhibition and revealed that different substitution groups greatly affect the inhibitory potency towards EcGUS, and meta-chloro-substitution was most beneficial for EcGUS inhibition among all substitution groups tested. The effects of substitution on furan or thiazolidin moiety worth further studies.

When Wallace et al.^32^ first confirmed that selectively inhibition of bacterial β-glucuronidases could alleviate CPT-11-induced toxicity in 2010, they also identified four uncompetitive EcGUS inhibitors. Scutellarein and caffeic acid ethyl ester were found to function as potent competitive inhibitors for EcGUS^28^^,^^33^. Our previous study found that sanggenon C and kuwanon G exhibited mixed-type inhibition against EcGUS, while amoxapine demonstrated uncompetitive inhibition against EcGUS^25^. In this study, we characterised the inhibition behaviours of compounds **1**, **2**, and **3**, and figured out that they were competitive EcGUS inhibitors. These findings demonstrated the complex interactions between EcGUS and the inhibitors, whose influence on the in vivo efficacy needs detailed analysis.

In conclusion, the present study identified 13 synthetic thiazolidin-2-cyanamide derivatives as potent EcGUS inhibitors and found that chloro and fluorine substitutions at the C4-position of the phenyl moiety of the furan are beneficial for EcGUS inhibition. Our findings are beneficial for developing more potent EcGUS inhibitors from thiazolidin-2-cyanamide derivatives and the in vivo efficacy of discovered EcGUS inhibitors also warrant further study.

## Supplementary Material

Supplemental Material
