# Supplementary material for: Thiazolidin-2-cyanamides derivatives as novel potent Escherichia coli β-glucuronidase inhibitors and their structure–inhibitory activity relationships
Source: J Enzyme Inhib Med Chem. 2020 Sep 14;35(1):1736–42. doi: 10.1080/14756366.2020.1816998 (PMC7534389; doi:10.1080/14756366.2020.1816998)
Supplement: Supplemental Material [file IENZ_A_1816998_SM6585.pdf]

Thiazolidin-2-cyanamides derivatives as novel potent *Escherichia coli* β-glucuronidase inhibitors and their structure-inhibitory activity relationships

Tao-Shun Zhou [1,#], Bin Wei [1,#], Min He [3,#], Ya-Sheng Li [1], Ya-Kun Wang [1], Si-Jia Wang [1,2], Jian-Wei Chen [1], Hua-Wei Zhang [1], Zi-Ning Cui [3,*], Hong Wang [1,*]

1 College of Pharmaceutical Science & Collaborative Innovation Center of Yangtze River Delta Region Green Pharmaceuticals, Zhejiang University of Technology, Hangzhou 310014, China; hongw@zjut.edu.cn (H. W.); Tel.: 86-571-8832-0622
2 Center for Human Nutrition, David Geffen School of Medicine, University of California, Rehabilitation Building 32-21, 1000 Veteran Avenue, Los Angeles, CA 90024, USA
3 State Key Laboratory for Conservation and Utilization of Subtropical Agro-bioresources, Lingnan Guangdong Laboratory of Modern Agriculture, Integrative Microbiology Research Centre, Guangdong Province Key Laboratory of Microbial Signals and Disease Control, South China Agricultural University, Guangzhou 510642, China

Supporting Information

Contents:

Page 2-6: Spectroscopic data of compounds 1-13

Spectroscopic data of compounds 1-13

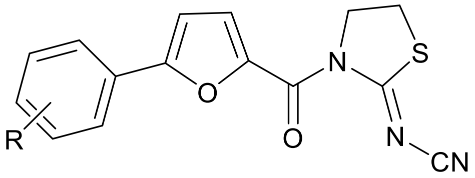

1 R= 2-Cl
2 R= 3-Cl
3 R= 4-Cl
4 R= 2-F
5 R= 4-F
6 R= 2,4-di-F
7 R= 2,6-di-F
8 R= 2-NO_2
9 R= 4- NO_2
10 R= 4-Br
11 R= 4-Me
12 R= 4-OMe
13 R= H

(*Z*)-*N*-(3-(5-(2-chlorophenyl) furan-2-carbonyl) thiazolidin-2-ylidene) cyanamide (1)

White solid: yield 62%. m.p. 101-102 ºC. ^{1}H NMR (400 MHz, DMSO-d_6) δ: 7.85 (d, *J* = 7.6 Hz, 1H, ArH), 7.64 (d, *J* = 8.1 Hz, 2H, ArH+Fu), 7.51 (dq, *J* = 15.4, 7.4 Hz, 2H, ArH), 7.34 (d, *J* = 2.9 Hz, 1H, FuH), 4.41 (t, *J* = 6.9 Hz, 2H, CH_2), 3.65 (t, *J* = 6.9 Hz, 2H, CH_2). ^{13}C NMR (100 MHz, DMSO-d_6) δ: 178.49 (C=O), 156.66 (C=N), 153.26 (C-5,Fu), 144.91 (C-2, Fu), 130.97 (C-2, Ar), 130.80 (C-1, Ar), 130.24 (C-4,Ar), 128.85 (C-3,Ar), 127.78 (C-6,Ar), 126.95 (C-5,Ar), 122.47 (C≡N), 114.54 (C-3, Fu), 113.17 (C-4, Fu), 53.04 (CH_2), 28.86 (CH_2). ESI-MS: *m/e* 332.1 $[M+H]^+$. Anal. Calcd. (%) for $C_{15}H_{10}ClN_3O_2S$: C, 54.30; H, 3.04; N, 12.67. Found: C, 54.13; H, 3.27; N, 12.40.

(*Z*)-*N*-(3-(5-(3-chlorophenyl) furan-2-carbonyl) thiazolidin-2-ylidene) cyanamide (2)

Light yellow solid: yield 61%. m.p. 166-167 ºC. ^{1}H NMR (400 MHz, DMSO-d_6) δ: 7.90 (s, 1H, ArH), 7.79 (d, *J* = 7.6 Hz, 1H, ArH), 7.63 (d, *J* = 3.0 Hz, 1H, Fu), 7.54 (dt, *J* = 16.8, 7.9 Hz, 2H, ArH), 7.39 (d, *J* = 3.2 Hz, 1H, FuH), 4.43 (t, *J* = 6.9 Hz, 2H, CH_2), 3.67 (t, *J* = 6.9 Hz, 2H,

CH_2). ^{13}C NMR (100 MHz, DMSO-*d*$_6$) δ: 178.48 (C=O), 156.52 (C=N), 155.37 (C-5, Fu), 145.09 (C-2, Fu), 133.99 (C-3, Ar), 131.05 (C-1, Ar), 130.50 (C-5, Ar), 129.18 (C-4, Ar), 124.26 (C-4, Ar), 123.31 (C-6, Ar), 123.07 (C≡N), 114.56 (C-3, Fu), 109.87 (C-4, Fu), 53.13 (CH_2), 28.87 (CH_2). ESI-MS: *m/e* 332.1 $[M+H]^+$. Anal. Calcd. (%) for $C_{15}H_{10}ClN_3O_2S$: C, 54.30; H, 3.04; N, 12.67. Found: C, 54.22; H, 2.91; N, 12.88.

(*Z*)-*N*-(3-(5-(4-chlorophenyl) furan-2-carbonyl) thiazolidin-2-ylidene) cyanamide (3)

White solid: yield 72%. m.p. 149-150 °C. ^{1}H NMR (400 MHz, DMSO-*d*$_6$) δ: 7.86 (d, *J* = 7.7 Hz, 2H, ArH), 7.60 (dd, *J* = 7.7 Hz, 3H, ArH+ FuH), 7.32 (d, *J* = 2.8 Hz, 1H, FuH), 4.42 (t, *J* = 6.8 Hz, 2H, CH_2), 3.66 (t, *J* = 6.8 Hz, 2H, CH_2).^{13}C NMR (100 MHz, DMSO-*d*$_6$) δ: 178.51 (C=O), 156.59 (C=N), 156.03 (C-5, Fu), 144.95 (C-2, Fu), 134.14 (C-4, Ar), 129.30 (C-3, Ar), 127.46 (C-1, Ar), 126.37 (C-2, Ar), 123.56 (C≡N), 114.64 (C-3, Fu), 109.34 (C-4, Fu), 53.19 (CH_2), 28.91(CH_2). ESI-MS: *m/e* 332.2 $[M+H]^+$. Anal. Calcd. (%) for $C_{15}H_{10}ClN_3O_2S$: C, 54.30; H, 3.04; N, 12.67. Found: C, 54.52; H, 3.21; N, 12.43.

(*Z*)-*N*-(3-(5-(2-fluorophenyl) furan-2-carbonyl) thiazolidin-2-ylidene) cyanamide (4)

Light yellow solid: yield 64%. m.p. 137-138 ºC. ^{1}H NMR (400 MHz, DMSO-*d*$_6$) δ: 7.85 (t, *J* = 7.6 Hz, 1H, ArH), 7.63 (d, *J* = 3.3 Hz, 1H, FuH), 7.50-7.55 (m, 1H, ArH), 7.38-7.44 (m, 2H, ArH), 7.12 (s, 1H, FuH), 4.42 (t, *J* = 6.9 Hz, 2H, CH_2), 3.66 (t, *J* = 6.9 Hz, 2H, CH_2). ^{13}C NMR (100 MHz, DMSO-*d*$_6$) δ: 178.49 (C=O), 158.61 (d, ^1J = 251.7 Hz, C-2, Ar), 156.57 (C=N), 151.24 (d, ^4J = 2.7 Hz, C-5, Fu), 144.74 (C-2, Fu), 131.38 (d, ^3J = 8.6 Hz, C-4, Ar), 126.65 (d, ^4J = 2.14 Hz, C-4, Fu), 125.25(d, ^4J = 3.36 Hz, C-5, Ar), 123.07 (C≡N), 116.66 (d, ^2J = 23.10 Hz, C-3, Ar), 116.50 (d, ^2J = 11.5 Hz, C-1, Ar), 114.53 (C-3, Fu), 112.36 (d, ^3J = 10.4 Hz, C-6, Ar), 53.09 (CH_2), 28.86 (CH_2). ESI-MS: *m/e* 316.2 $[M+H]^+$. Anal. Calcd. (%) for $C_{15}H_{10}FN_3O_2S$: C, 57.14; H, 3.20; N, 13.33. Found: C, 56.92; H, 3.29; N, 13.56.

(*Z*)-*N*-(3-(5-(4-fluorophenyl) furan-2-carbonyl) thiazolidin-2-ylidene) cyanamide (5)

Light yellow solid: yield 72%. m.p. 129-130 ºC. ^{1}H NMR (400 MHz, DMSO-*d*$_6$) δ: 7.95 –7.84 (m, 2H, ArH), 7.62 (s, 1H, FuH), 7.38 (t, *J* = 8.3 Hz, 2H, ArH), 7.26 (s, 1H, FuH), 4.42 (t, *J* = 6.8 Hz, 2H, CH_2), 3.66 (t, *J* = 6.8 Hz, 2H, CH_2). ^{13}C NMR (100 MHz, DMSO-*d*$_6$) δ: 178.45 (C=O), 162.64 (d, ^1J = 247.9 Hz, C-4, Ar), 156.55 (C=N), 156.35 (C-5, Fu), 144.69 (C-2, Fu),

127.02 (d, ^3J = 8.6 Hz, C-2, C-6, Ar), 125.27 (d, ^4J = 3.1 Hz, C-1, Ar), 123.67 (C≡N), 116.29 (d, ^2J = 22.2 Hz, C-3, C-5, Ar), 114.64(C-3, Fu), 108.57(C-2, Fu), 53.15 (CH_2), 28.86 (CH_2). ESI-MS: *m/e* 316.2 $[M+H]^+$. Anal. Calcd. (%) for $C_{15}H_{10}FN_3O_2S$: C, 57.14; H, 3.20; N, 13.33. Found: C, 57.33; H, 2.99; N, 13.52.

(Z)-N-(3-(5-(2, 4-difluorophenyl) furan-2-carbonyl) thiazolidin-2-ylidene) cyanamide (6)

White solid: yield 69%. m.p. 140-141 ºC. ^{1}H NMR (400 MHz, DMSO-d_6) δ: 7.89 (q, *J* = 7.6 Hz, 1H, ArH), 7.62 (s, 1H, FuH), 7.56 – 7.47 (m, 1H, ArH), 7.32 (t, *J* = 8.5 Hz, 1H, ArH), 7.09 (s, 1H, FuH), 4.41 (t, *J* = 6.8 Hz, 2H, CH_2), 3.65 (t, *J* = 6.8 Hz, 2H, CH_2). ^{13}C NMR (100 MHz, DMSO-d_6) δ: 178.61 (C=O), 162.66 (dd, ^1J = 250.6, ^3J = 12.4 Hz, C-4, Ar), 159.01 (dd, ^1J = 254.5, ^3J = 12.6 Hz, C-2, Ar), 156.64 (C=N), 150.66 (d, ^4J = 2.7 Hz, C-5, Fu), 144.86 (C-2, Fu), 128.30 (dd, ^3J = 10.1, ^4J = 3.8 Hz, C-6, Ar), 123.15 (C≡N) , 114.61 (C-3, Fu) , 113.72 (dd, ^3J = 12.0, ^4J = 3.8 Hz, C-1, Ar), 112.86 (dd, ^2J = 22.0, ^4J = 3.4 Hz, C-5, Ar), 111.98 (d, ^3J = 9.8 Hz, C-4, Fu), 105.38 (d, ^2J = 26.0 Hz, ^2J = 26.1 Hz, C-3, Ar), 53.17 (CH_2), 28.94 (CH_2). ESI-MS: *m/e* 334.2 $[M+H]^+$. Anal. Calcd. (%) for $C_{15}H_9F_2N_3O_2S$: C, 54.05; H, 2.72; N, 12.61. Found: C, 54.21; H, 2.54; N, 12.80.

(*Z*)-*N*-(3-(5-(2, 6-difluorophenyl) furan-2-carbonyl) thiazolidin-2-ylidene) cyanamide (7)

Yellow solid: yield 69%. m.p. 136-137 ºC. ^{1}H NMR (400 MHz, DMSO-d_6) δ: 7.67 – 7.52 (m, 2H, FuH + ArH), 7.32 (t, *J* = 9.3 Hz, 2H, ArH), 7.12 (s, 1H, FuH), 4.40 (t, *J* = 6.8 Hz, 2H, CH_2), 3.64 (t, *J* = 6.8 Hz, 2H, CH_2). ^{13}C NMR (100 MHz, DMSO-d_6) δ: 178.26 (C=O), 158.93 (dd, ^1J = 253.7, ^3J = 6.2 Hz, C-2, C-6, Ar), 156.71 (C=N), 146.43(C-5, Fu), 145.58(C-2, Fu), 131.92 (dd, ^3J = 10.7 Hz, ^3J = 10.7 Hz, Ar), 121.79 (C≡N), 114.93 (dd, ^4J = 5.5 Hz, ^4J = 5.4 Hz, C-4, Fu), 114.46 (C-3, Fu), 112.61 (dd, ^2J = 24.9 Hz, C-3, C-5, Ar), 106.84 (dd, ^2J = 15.95, ^2J = 16.02 Hz, C-1, Ar), 52.87 (CH_2), 28.80 (CH_2). ESI-MS: *m/e* 334.1 $[M+H]^+$. Anal. Calcd. (%) for $C_{15}H_9F_2N_3O_2S$: C, 54.05; H, 2.72; N, 12.61. Found: C, 53.89; H, 2.95; N, 12.74.

(*Z*)-*N*-(3-(5-(2-nitrophenyl) furan-2-carbonyl) thiazolidin-2-ylidene) cyanamide (8)

Yellow solid: yield 75%. m.p. 135-136 °C. ^{1}H NMR (400 MHz, DMSO-d_6) δ: 8.00 (d, *J* = 8.0 Hz, 1H, ArH), 7.95 (d, *J* = 7.8 Hz, 1H, ArH), 7.83 (t, *J* = 7.6 Hz, 1H, ArH), 7.72 (t, *J* = 7.6 Hz, 1H, ArH), 7.62 (d, *J* = 3.2Hz, 1H, FuH), 7.19 (d, *J* = 3.1 Hz, 1H, FuH), 4.38 (t, *J* = 6.8 Hz, 2H,

CH_2), 3.62 (t, *J* = 6.8 Hz, 2H, CH_2). ^{13}C NMR (100 MHz, DMSO-d_6) δ: 178.57 (C=O), 156.49 (C=N), 151.90 (C-5, Fu), 147.20 (C-2, Ar), 146.02 (C-2, Fu), 132.67 (C-1, Ar), 130.89 (C-4, Ar), 129.63 (C-5, Ar), 124.14 (C-3, Ar), 122.51 (C-5, Ar), 121.26 (C≡N), 114.47 (C-3, Fu), 112.21 (C-4, Fu), 52.98 (CH_2), 28.86(-CH_2). ESI-MS: *m/e* 343.2 $[M+H]^+$. Anal. Calcd. (%) for $C_{15}H_{10}N_4O_4S$: C, 52.63; H, 2.94; N, 16.37. Found: C, 52.81 ; H, 3.10; N, 16.12.

(*Z*)-*N*-(3-(5-(4-nitrophenyl) furan-2-carbonyl) thiazolidin-2-ylidene) cyanamide (9)

Yellow solid: yield 80%. m.p. >290 ºC. ^1H NMR (600 MHz, DMSO-d_6) δ: 8.35 (d, *J* = 8.8 Hz, 2H, ArH), 8.07 (d, *J* = 8.8 Hz, 2H, ArH), 7.65 (d, *J* = 3.8 Hz, 1H, FuH), 7.54 (d, *J* = 3.8 Hz, 1H, FuH), 4.45 (t, *J* = 7.0 Hz, 2H, CH_2), 3.68 (t, *J* = 7.0 Hz, 2H, CH_2). ^{13}C NMR (150 MHz, DMSO-d_6) δ: 178.64 (C=O), 156.62 (C=N), 154.51 (C-5, Fu), 147.27 (C-4, Ar), 146.14 (C-2, Fu), 134.25 (C-1, Ar), 125.49 (C-3, C-5, Ar), 124.52 (C-2, C-6, Ar), 123.10 (C≡N), 114.51 (C-3, Fu), 112.04 (C-4, Fu), 53.16 (CH_2), 28.90 (CH_2). ESI-MS: *m/e* 343.3 $[M+H]^+$. Anal. Calcd. (%) for $C_{15}H_{10}N_4O_4S$: C, 52.63; H, 2.94; N, 16.37. Found: C, 52.86; H, 3.10; N, 16.11.

(*Z*)-*N*-(3-(5-(4-bromophenyl) furan-2-carbonyl) thiazolidin-2-ylidene) cyanamide (10)

White solid: yield 77%. m.p. 169-170 ºC. ^1H NMR (400 MHz, DMSO-d_6) δ: 7.78 (d, *J* = 8.0 Hz, 2H, ArH), 7.72 (d, *J* = 8.0 Hz, 2H, ArH), 7.62 (d, *J* = 3.0 Hz, 1H, FuH), 7.32 (d, *J* = 3.1 Hz, 1H, FuH), 4.44 (t, *J* = 6.8 Hz, 2H, CH_2), 3.67 (t, *J* = 6.8 Hz, 2H, CH_2). ^{13}C NMR (100 MHz, DMSO-d_6) δ: 178.41 (C=O), 156.52(C=N), 156.05 (C-5, Fu), 144.89 (C-2, Fu), 132.11 (C-3, C-5, Ar), 127.72 (C-1, Ar), 126.47 (C-4, Ar), 123.50 (C-2, C-6, Ar), 122.82 (C≡N), 114.59 (C-3, Fu), 109.29 (C-4, Fu), 53.14(CH_2), 28.85(CH_2). ESI-MS: *m/e* 376.1 $[M+H]^+$. Anal. Calcd. (%) for $C_{15}H_{10}BrN_3O_2S$: C, 47.89; H, 2.68; N, 11.17. Found: C, 48.04; H, 2.83 ; N, 10.98.

(*Z*)-*N*-(3-(5-(p-tolyl) furan-2-carbonyl) thiazolidin-2-ylidene) cyanamide (11)

Light yellow solid: yield 80%. m.p. 151-152 ºC. ^1H NMR (400 MHz, DMSO-d_6) δ: 7.73 (d, *J* = 7.6 Hz, 2H, ArH), 7.64 – 7.56 (m, 1H, FuH), 7.33 (d, *J* = 7.7 Hz, 2H, ArH), 7.23 – 7.16 (m, 1H, FuH), 4.41 (t, *J* = 6.9 Hz, 2H, CH_2), 3.65 (t, *J* = 6.9 Hz, 2H, CH_2), 2.36 (s, 3H, CH_3). ^{13}C NMR (100 MHz, DMSO-d_6) δ: 178.36 (C=O), 157.62(C=N), 156.52(C-5, Fu), 144.31 (C-2, Fu), 139.45 (C-4, Fu), 129.69 (C-3, C-5, Ar), 125.89 (C-1, Ar), 124.63 (C-2, C-6, Ar), 123.82

(C≡N), 114.67 (C-3, Fu), 108.04 (C-4, Fu), 53.14 (CH_2), 28.83 (CH_2), 20.90 (CH_3). ESI-MS: *m/e* 312.2 $[M+H]^+$. Anal. Calcd. (%) for $C_{16}H_{13}N_3O_2S$: C, 61.72; H, 4.21; N, 13.50. Found: C, 61.87; H, 4.43; N, 13.36.

(*Z*)-*N*-(3-(5-(4-methoxyphenyl) furan-2-carbonyl) thiazolidin-2-ylidene) cyanamide (12)

White solid: yield 66%. m.p. 151-152 ºC. ^1H NMR (600 MHz, DMSO-d_6) δ: 7.78 (d, J = 8.8 Hz, 2H, ArH), 7.60 (d, J = 3.8 Hz, 1H, FuH), 7.13 (d, J = 3.8 Hz, 1H, FuH), 7.09 (d, J = 8.9 Hz, 2H, ArH), 4.40 (t, J = 7.0 Hz, 2H, CH_2), 3.83 (s, 3H, CH_3), 3.65 (t, J = 7.0 Hz, 2H, CH_2). ^{13}C NMR (150 MHz, DMSO-d_6) δ: 178.38 (C=O), 160.43 (C=N), 157.87 (C-4, Ar), 156.53 (C-5, Fu), 144.07 (C-2, Fu), 126.49 (C-1, Ar), 124.23 (C-2, C-6, Ar), 121.32 (C≡N), 114.78 (C-3, Fu), 114.70 (C-3, C-5, Ar), 107.27 (C-4, Fu), 55.36 (CH_3O), 53.21 (CH_2), 28.89 (CH_2). ESI-MS: *m/e* 328.3 $[M+H]^+$. Anal. Calcd. (%) for $C_{16}H_{13}N_3O_3S$: C, 58.70; H, 4.00; N, 12.84. Found: C, 58.56; H, 3.79; N, 12.96.

(*Z*)-*N*-(3-(5-phenylfuran-2-carbonyl) thiazolidin-2-ylidene) cyanamide (13)

Light yellow solid: yield 70%. m.p. 143-144 ºC. ^1H NMR (400 MHz, DMSO-d_6) δ: 7.85 (d, J = 7.6 Hz, 2H, ArH), 7.62 (s, 1H, FuH), 7.53 (t, J = 7.4 Hz, 2H, ArH), 7.49 – 7.42 (m, 1H, ArH), 7.27 (s, 1H, FuH), 4.43 (t, J = 6.8 Hz, 2H, CH_2), 3.66 (t, J = 6.8 Hz, 2H, CH_2). ^{13}CNMR (100 MHz, DMSO-d_6) δ: 178.41 (C=O), 157.22 (C=N), 156.56 (C-5, Fu), 144.65 (C-2, Fu), 129.54 (C-1, Ar), 129.13 (C-3, C-5, Ar), 128.54 (C-4, Ar), 124.64 (C-2, C-6, Ar), 123.60 (C≡N), 114.64 (C-3, Fu), 108.68 (C-4, Fu), 53.13 (CH_2), 28.85(CH_2). ESI-MS: *m/e* 298.3 $[M+H]^+$. Anal. Calcd. (%) for $C_{15}H_{11}N_3O_2S$: C, 60.59; H, 3.73; N, 14.13. Found: C, 60.73, H, 3.51; N, 14.28.
